# Conceptualization of relative size by honeybees

**DOI:** 10.3389/fnbeh.2014.00080

**Published:** 2014-03-14

**Authors:** Aurore Avarguès-Weber, Daniele d’Amaro, Marita Metzler, Adrian G. Dyer

**Affiliations:** ^1^Biological and Experimental Psychology, School of Biological and Chemical Sciences, Queen Mary University LondonLondon, UK; ^2^Institut für Zoologie III (Neurobiologie), Johannes Gutenberg-UniversitätMainz, Germany; ^3^Department of Physiology, Monash UniversityClayton, VIC, Australia; ^4^School of Media and Communication, Royal Melbourne Institute of TechnologyMelbourne, VIC, Australia

**Keywords:** relational concept learning, relative size, long wavelength photoreceptor, honeybee, *Apis mellifera*

## Abstract

The ability to process visual information using relational rules allows for decisions independent of the specific physical attributes of individual stimuli. Until recently, the manipulation of relational concepts was considered as a prerogative of large mammalian brains. Here we show that individual free flying honeybees can learn to use size relationship rules to choose either the larger or smaller stimulus as the correct solution in a given context, and subsequently apply the learnt rule to novel colors and shapes providing that there is sufficient input to the long wavelength (green) photoreceptor channel. Our results add a novel, size-based conceptual rule to the set of relational concepts that honeybees have been shown to master and underline the value of bees as an animal model for studying the emergence of conceptualization abilities.

## Introduction

Materializing abstract relations between objects into meaningful labels is considered to be at the cornerstone of human cognition (Murphy, 2002; Doumas et al., 2008; Halford et al., 2010; Mareschal et al., 2010). Human children learn the ability to manipulate concepts of relations (e.g., “same”, “above”, “bigger than”) through multiple comparisons occurring during language acquisition. Relational concepts should indeed be independent of the specific objects linked by a learnt rule (Zentall et al., 2002, 2008). Such ability has long been considered as a prerogative of the primates brain. Conceptualization capacity takes a long time to develop in a primate brain, and neurobiological correlates of concept learning have been localized at the level of the prefrontal cortex (Wallis et al., 2001; Miller et al., 2003). Yet, the capacity to elaborate relational concepts exists in some non-primate vertebrates such as dolphins (Mercado et al., 2000; Kilian et al., 2003) and birds (Pepperberg, 1987; Blaisdell and Cook, 2005; Katz and Wright, 2006; Scarf et al., 2011; Suková et al., 2013), demonstrating that different neural organization can support conceptualization abilities.

The miniature brain of the honeybee has also recently been shown to be capable of extracting the constant relations existing in a set of variable visual stimuli and of using the acquired information as a rule in subsequent choices of novel stimuli (for review see Zhang, 2006; Avarguès-Weber and Giurfa, 2013). Impressively, honeybees succeeded in acquiring concepts such as “same”/“different” or “above”/“below” (Giurfa et al., 2001; Avarguès-Weber et al., 2011b; Perry and Barron, 2013). In addition, the bees were able to simultaneously combine two concepts to solve novel problems (Avarguès-Weber et al., 2012). The demonstration of concept learning in bees has consequently raised new questions on the minimal complexity of the neural networks required to allow conceptualization abilities.

Honeybees are central-place foragers that often face challenging visual problems to successfully travel back and forth between the hive and various flower patches that may be separated by long distances (Pahl et al., 2011; Galizia et al., 2012). Their navigation abilities do not only include the use of a sky-based compass (Rossel and Wehner, 1986) and landmark learning (Cartwright and Collett, 1982, 1983; Collett, 1996) but benefit also from memorizing relations between different landmarks, potentially as the basis of a cognitive-map like internal representation of the foraging environment (Menzel et al., 2005; Avarguès-Weber and Giurfa, 2013; Dyer and Rosa, 2013). The bees also rely on flexible visual pattern recognition strategies to discriminate and classify the most profitable flowers from which to collect nutrition (Srinivasan, 2010; Avarguès-Weber et al., 2011a). In particular, they categorize visual objects such as landmarks or flowers based on common features and can integrate such features into generic configurations facilitating reliable object recognition, independently of viewpoint or illumination changes (Stach et al., 2004; Zhang et al., 2004; Dyer and Vuong, 2008; Avarguès-Weber et al., 2010b; Dyer and Griffiths, 2012).

For a flying insect, the absolute size of an object like a flower may not be a reliable cue for identifying a target as the size of an object varies with the actual viewing distance during an approach flight (Giurfa et al., 1996). However, the difference in relative size between simultaneously or sequentially presented flowers or landmarks could potentially be of high value for independent decision making if bees possess a capacity to judge objects based on a relative size rule such as “smaller than” or “larger than”.

In the current study, we investigated whether the honeybees possess the faculty to learn a rule based on the relative size between different stimuli, and then to potentially transfer the rule to novel visual stimuli. To this end, free-flying bees were trained in a learning situation in which only relative size differences could predict reward/punishment outcomes. We also studied the involvement of the green color channel in the conceptualization task by modulating the L-receptor contrast. Honeybees possess three photoreceptor types (S/UV, M/Blue and L/Green) (Peitsch et al., 1992) and the achromatic properties of objects (orientation, stimulus contours) are indeed processed by the L-receptor alone (Giger and Srinivasan, 1996; Hempel de Ibarra et al., 2002; Hempel de Ibarra and Giurfa, 2003; Stach et al., 2004).

## Materials and methods

### Apparatus

Experiments were conducted with individually tagged and tested honeybees (*Apis mellifera L*.) trained to freely visit the experimental apparatus, a 50 cm diameter vertical screen which could be rotated to vary the spatial arrangement of the stimuli presented on it (see Dyer et al., 2005; Dyer and Vuong, 2008). Only one bee was present at a time at the apparatus during the training and the tests. Four stimuli (two identical S+ and two identical S− stimuli) were presented simultaneously on top of landing platforms offering a 10 μL drop of either a 25% sucrose solution (S+) or a 60 mM quinine hemisulfate solution (S−), which promotes enhanced visual discrimination performances (Avarguès-Weber et al., 2010a). The stimuli were attached on freely rotating 6 × 8 cm hangers that could be positioned in a number of random spatial positions and rearranged during the training by a rotation of the whole screen or manual displacements of the hangers (Dyer et al., 2005). Care was taken to arrange the stimuli in a pseudorandom manner on the presentation screen to avoid the use of spurious information not relevant to the task. Stimuli and landing platforms were washed with ethanol between foraging bouts and before the tests.

### Stimuli

**Figure 1:**
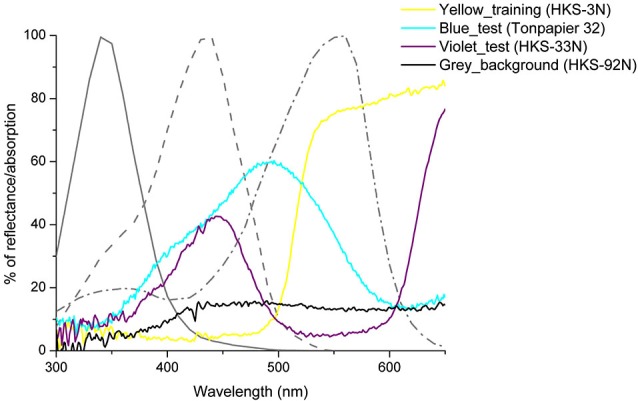
**Spectral reflection curves of the stimuli used in our experiments and relative absorptions of the three honeybee’s photoreceptors, S, M and L (shown in gray)**. The colors distances between these colors in two perceptual spaces proposed for the honey bee, the color hexagon (Chittka, 1992) and the color opponent coding space (Backhaus, 1991) were high enough as to allow discrimination in all cases (Dyer et al., 2008; see Table 1).

Training stimuli were either squares or diamonds (45° rotated squares), varying in size, cut from HKS-3N (K + E Stuttgart, Stuttgart–Feuerbach, Germany) human-yellow paper presented on a HKS-92N human-gray background (Figure 1). The size of the stimuli edges varied from 1 to 6 cm with a 1 cm step between stimulus alternatives, thus yielding six possible sizes whose areas increased from 1 to 36 cm^2^. Prior testing established that bees can discriminate 3 × 3 vs. 4 × 4 cm yellow square stimuli differing only by a edge-size difference of 1 cm at a level of 70.7 ± 2.2% correct choices in a non-reinforced learning test following a 10 choices differential conditioning protocol (sucrose vs. quinine). Adjacent stimuli in the size scale could thus be discriminated in our experimental setup.

### Training and test procedures

**Figure 2:**
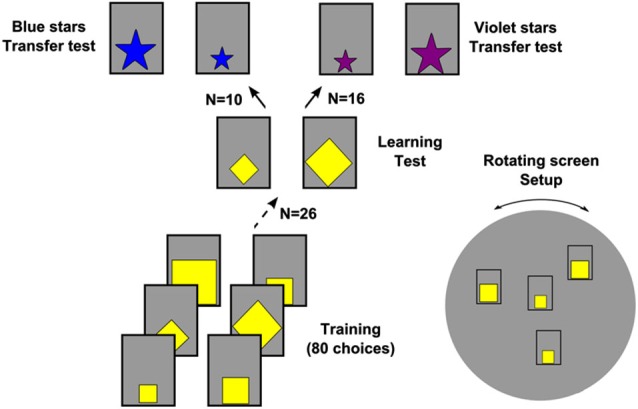
**Training and testing procedure**. The bees had to choose between stimuli varying only in size. At each foraging bout, the bees faced stimuli of two different sizes taken randomly between the six available sizes (from 1 to 6 cm with 1 cm step). Depending on testing group, the smallest or the largest stimuli were rewarded with a sucrose solution while the alternative stimuli were associated with a quinine solution. Each stimulus was presented twice. Four stimuli (two identical S+ and two identical S−) were then simultaneously offered to the bees. During training, stimuli shapes and size combinations varied to facilitate concept learning. Once training was completed, the bees were subjected to non-rewarded transfer tests intermingled with refreshing training trials. Note that only a subset of all possible tests was proposed to each bee (see text for details). The insert show a representation of the rotating screen on which the four stimuli are vertically presented in a random spatial organization (Dyer et al., 2005).

The training phase consisted of 80 choices (landings on a stimulus platform). A typical foraging bout (before the bee returned to the hive) consisted of 4–6 choices so that to complete the training, bees needed from 13 to 20 foraging bouts. Within a foraging bout, the S+ and S− were identical geometrical shapes differing only by their size (Figure 2). One group of bees (*n* = 13) was trained to choose the rewarded larger stimulus and to avoid the penalized smaller stimulus, while another group of bees (*n* = 13) was trained with the reversed rewarding contingency, i.e., to choose the rewarded smaller stimulus and to avoid the penalized larger stimulus.

Both the stimuli size and shape (square or diamond) were varied between foraging bouts in a pseudo random in order to facilitate rule learning as only the relative size criterion (“smaller” or “larger”) remained predictive of the reinforcement outcome (Zentall et al., 2002, 2008; Avarguès-Weber and Giurfa, 2013). All 15 possible combinations of sizes (6 different sizes from 1 × 1 to 6 × 6 cm) were presented to the bees in a pseudo random order.

Importantly, this experimental design implied that a given stimulus size (e.g., 3 × 3 cm) could be either rewarded or punished in different foraging bouts depending on the alternative size with which it was presented (e.g., 2 × 2 or 5 × 5 cm) (Figure 2). Only the two extremes of the scale, 1 × 1 and 6 × 6 cm, had a constant association with reinforcement, i.e., bee trained for “larger than” had variable reinforcements in all stimuli except for 6 × 6 cm stimuli which were always rewarded and for 1 × 1 cm stimuli which were always punished with the quinine solution; in the case of bees trained for “smaller than”, 1 × 1 cm stimuli were always rewarded and 6 × 6 cm stimuli were always punished. The performance during training was analyzed in terms of the proportion of correct landings within blocks of 10 consecutive choices.

After completion of the training phase, the bees (*n* = 26) were subjected to two types of non-reinforced tests. First, a *learning test* was performed in which the bees faced either square or diamond yellow stimuli, 2.5 × 2.5 vs. 4.5 × 4.5 cm in size (Figure 2). Despite the fact that the same shapes and color were used during the training, none of these sizes was presented before. Bees trained to choose the relative smaller stimuli should prefer the 2.5 × 2.5 cm stimulus while those trained to choose the relative larger stimuli should prefer the 4.5 × 4.5 cm stimulus. In choosing these sizes, we took care to avoid that the last rewarded stimulus during the training (smaller or larger) was adjacent in absolute size to the correct test stimulus.

After the learning test, a *transfer test* was performed in which the bees were presented with five point stars 2.5 × 2.5 vs. 4.5 × 4.5 cm in size and which were either human-blue (Tonpapier no. 32 Baehr, Germany) for a subgroup of bees (*n* = 10) or human-violet (HKS-33N) for another subgroup of bees (*n* = 16) (Figure 2). The surface areas of the star shapes were respectively 2 cm^2^ and 6.4 cm^2^. Thus, in the transfer test, bees were confronted with novel sizes, novel shapes and novel colors.

During the tests, the first twenty choices of the test bee were recorded. Two refreshing foraging bouts presenting the training conditions were intermingled between the non-reinforced tests.

### Photoreceptor contrast and relative intensity of colors

The blue and the violet colors used in the transfer tests were chosen in order to manipulate the receptor specific contrasts provided by our stimuli with respect to the gray background on which they were presented (see Table 1).

**Table 1:** **Photoreceptor contrasts, relative intensity and color distances of the stimuli**.

	Yellow (HKS-3N)	Blue (tonpapier-32)	Violet (HKS-33N)
UV-Receptor (Short Wavelengths)	0.88	3.03	2.44
Blue-Receptor (Medium Wavelengths)	0.43	3.26	2.37
Green-Receptor (Long Wavelengths)	3.62	2.98	0.88
Relative Intensity	4.93	9.27	5.69
Color Distance (Hexagon units)	Bkgd.: 0.42 Blue: 0.44 Violet: 0.65	Bkgd.: 0.06 Violet: 0.23	Bkgd.: 0.18
Color Distance (COC units)	Bkgd.: 8.73 Blue: 9.16 Violet: 11.68	Bkgd.: 0.42 Violet: 3.87	Bkgd.: 4.09

Receptor-specific contrasts, i.e., the relative number of absorbed quanta of light *q* with respect to the background, were calculated as:

$$\begin{array}{l} q_{i}=\int_{300}^{650}Si(\lambda )R(\lambda )I(\lambda )d\lambda /Si(\lambda )R(\lambda )I(\lambda )d\lambda \\ \;i\;=\;\mathrm{uv},\;\mathrm{blue},\;\mathrm{green}\;\mathrm{receptor} \end{array}$$

with *I*(λ) being the spectral intensity distribution of the illuminating light (norm light function 6500 K daylight (D65)), *R*(λ) the spectral reflectance of the stimulus, *B*(λ) the spectral reflectance of the background and *Si*(λ) the spectral sensitivity of the bee receptor with index *i* (Peitsch et al., 1992). If values are close to 1, then the color offers poor achromatic contrast against the background for the specific receptor considered.

The relative intensity of a given stimulus was calculated as the sum of the receptor contrasts relative to the background.

Chromatic contrasts (color distances) between stimuli (S) or between a stimulus and the background (Bkgd.) was calculated using two different color spaces, the color opponent coding space (Backhaus, 1991) and the color hexagon (Chittka, 1992).

Table 1 shows the receptor-specific contrasts, relative intensity values and color distances for all stimuli used in our experiments.

### Statistics

Learning curves were analyzed by means of ANOVA for repeated measurements to detect significant variations along the 8 blocks of 10 choices. Performances during the tests were analyzed by means of a one-sample *t*-test which allowed comparing the actual performance of the bees with a theoretical proportion of 50% (random choices). Test performance of two independent groups was compared by means of a two-sample *t*-test. Alpha was set at 0.05 in all cases.

## Results

**Figure 3:**
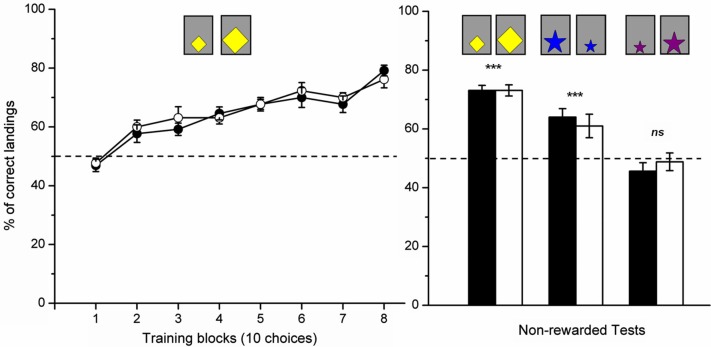
**Performance of the tested honeybees**. Percentage of correct choices in the training (left panel) and tests (right panel) phases. Performance of the bees rewarded on the larger stimuli are presented in black while performance of the bees rewarded on the smaller stimuli are presented in white. The dashed line indicates chance level performance. Data shown are means +s.e.m. *n* = 26 for the acquisition curve and learning test. 10 of the 26 bees were tested in the “blue stars transfer test” while the others were tested in the “violet stars transfer test” (***: *p* < 0.001; n.s: *p* > 0.05).

Bees trained to choose the smaller target improved their performance, irrespectively of the pseudo random variation of target sizes. Similarly, bees trained to choose the larger target also improved their performance along the training blocks (Figure 3, left panel). The increase in the percentage of correct choices along the 8 training blocks of 10 choices was significant (*n* = 26, repeated measures ANOVA, *F*_7,168_ = 29.7, *p* < 0.001; Figure 3, left panel), irrespectively of the relative size rewarded (*F*_7,168_ = 0.47, *p* = 0.85).

In the learning test, the bees rewarded for choosing the larger stimuli chose the novel larger stimulus in 73.1 ± 1.7% (mean ± SEM) of their choices, while the bees rewarded for choosing the smaller stimuli chose the novel smaller stimulus in 73.1 ± 1.9% of the choices. As there were no significant differences between both groups (two-sample *t*-test: *t*_24_ = 0.90, *p* = 0.38), performances in the learning test were pooled and shown to be significantly above chance level (pooled data: *n* = 26; one-sample *t*-test against 50% chance level: *t*_25_ = 18.57, *p* < 0.001; Figure 3, right panel).

In the second transfer test, the bees were subdivided in two subsets. The subset of bees (*n* = 10) that was tested with the blue-star stimuli were capable of transferring the learnt relational rule to the novel situation, which presented stimuli that differed both in shape and color from the training stimuli (Figure 2). In this test, the bees rewarded on the relatively larger yellow stimuli significantly preferred the larger blue star in 64.0 ± 2.9% of the choices, while the bees rewarded on the relatively smaller yellow stimuli preferred the smaller blue star in 61.0 ± 4.0% of the choices. Results were not significantly influenced by the training group (*t*_8_ = 0.6, *p* = 0.56) so that data from both groups could be pooled. The pooled performance was significantly different from chance expectation (*t*_9_ = 5.2, *p* < 0.001; Figure 3, right panel), thus demonstrating the faculty of bees to transfer the learnt relation to stimuli differing both in shape and color from the training stimuli.

The other subset of bees (*n* = 16) that was presented with violet-star stimuli failed to recognize the larger or smaller stimulus in the transfer test. Thus, the bees rewarded on the relatively larger yellow stimuli showed no significant preference for the larger violet star which they chose in 45.6 ± 2.9% of the choices, while the bees rewarded on the relatively smaller yellow stimuli chose the smaller violet star in 48.8 ± 3.0% of the choices. As there was no significant difference between both groups (*t*_14_ = 0.8, *p* = 0.46), performances were pooled. The resulting data did not differ significantly from a random choice (*t*_15_ = 1.4, *p* = 0.19; Figure 3, right panel), showing that this specific test situation did not allow for transfer of the learnt rule.

In order to determine why the transfer test was possible in the case of the blue stars but not in the case of the violet stars, a closer look at the chromatic and achromatic cues provided by these two colors is necessary. Table 1 shows that compared to the blue color, the violet color did not present a significant L-receptor contrast with respect to the background. From the other parameters considered, the S-receptor contrast, the M-receptor contrasts and the overall intensity can be excluded as relevant for the task because the yellow cardboard used to train and test the bees (learning test) allowed efficient learning and transfer despite its relatively low overall intensity, S-receptor and M-receptor contrasts. The chromatic contrast to the background can also be excluded as the factor explaining why transfer did not occur in the case of the violet stimuli. Indeed, this contrast was higher in the case of the violet stimuli than in the case of the blue ones which allowed a successful transfer of the learnt relation. Thus, L-receptor contrast, decreased in the case of violet stimuli, could account for the absence of transfer with the violet stars (see Table 1 for details).

## Discussion

Our results indicate that the honeybee is able to learn a conceptual rule based on the relations “smaller than” or “larger than”, irrespectively of the stimuli used. In our experiments, bees learned to choose either relatively smaller or larger closed shapes as the correct rewarding stimuli, even when the reward/punishment contingency of particular stimuli was changed in a pseudo random order. Furthermore, the bees could transfer such acquired knowledge to novel stimuli of different color and shape if these provided sufficient L-receptor contrast. We interpret these findings as an evidence of conceptual learning given that as our training regime used many variable stimuli leaving only relative size as the unique criterion predicting reinforcement outcomes. Concept learning was favored by pseudo-random stimulus variation (both in shape and size) along training and the transfer tests allowed us to exclude alternative low-level mechanisms (Avarguès-Weber and Giurfa, 2013).

### Conceptual learning vs. associative learning

A concept of size would allow to classify stimuli in terms of relative size differences along a size scale (A > B > C > D > E). This capacity would be in agreement with the ability to build partial stimulus hierarchies based on transitive relationships (Benard and Giurfa, 2004). Transitive inferences in bees are partial because their building of stimulus hierarchies is disturbed by recency effects which promotes more responding to the last rewarded stimulus (Benard and Giurfa, 2004). In our experiments we took care to present stimuli in a pseudo random sequence so that the last rewarded stimulus during the training (smaller or larger) was not adjacent in its absolute size to that of the correct transfer stimulus. We aimed, in this way, at depriving the bees of absolute size cues, which could have biased their choice based on a recency effect.

An additional potential problem of our experimental schedule is also common to transitive-inference experiments in which the training stimulus on top of the hierarchy (A) is always reinforced and the training stimulus at the bottom of the hierarchy (E) is always non-reinforced, i.e., A+ vs. B−, B+ vs. C−, C+ vs. D−, D+ vs. E−. For this reason, transitive inferences are tested by confronting stimuli that are equally reinforcing and non-reinforcing during training and that are never shown together (e.g., B vs. D). Although our protocol and the transitive-inference one are clearly different, they have in common the fact that the two extremes of the training scale are non-ambiguous in terms of their association with reinforcement. In our experiments, the group trained to the concept of “smaller than” experienced that the smallest stimulus (1 × 1 cm) was always positively reinforced and the largest stimulus (6 × 6 cm) was always negatively reinforced. The group trained to the concept of “larger than” experienced the opposite contingencies for the smallest and the largest stimuli. This may induce a potential problem for the transfer tests.

Indeed, for bees trained to always choose the smaller stimulus, the training stimuli that were adjacent to the appropriate test alternative 2.5 × 2.5 were 2 × 2 and 3 × 3. These stimuli were rewarded in 80% and 60% of their appearances during training. In contrast, the training stimuli that were adjacent to the inappropriate test alternative 4.5 × 4.5 were 4 × 4 and 5 × 5. These stimuli were rewarded in 40% and 20% of their appearances during training. Thus, it could be possible that bees chose the smaller test stimulus 2.5 × 2.5 because of its higher probabilistic association with reward. The same kind of reasoning could be applied for bees rewarded for choosing always the larger stimulus.

Yet, we think that it is unlikely that the bees solved the relative-size classification task using a strategy based on simple associative mechanisms based on the reinforcement history of each training stimulus. Although the bees could in theory learn the reward contingency of all absolute sizes (certain sizes being either positively or negatively reinforced in a probabilistic way depending on the alternative stimulus size) and then generalize the acquired associative strength toward the novel sizes used in tests, such a learning capacity would not only be memory demanding but hasn’t been, to our knowledge, demonstrated in insects.

Thus, although we cannot discard definitively an associative explanation for our results, we feel that in the light of previous findings proving concept formation in bees (see Avarguès-Weber and Giurfa, 2013 for review) it is safe to suggest that bees solved the problem by using a “smaller than” or a “larger than” conceptual rule. We acknowledge that further experiments are necessary to determine whether bees could, for instance, extrapolate the relative size concept acquired during training to novel sizes outside the learning scale. This experiment would be a clearer demonstration of conceptual learning as it would imply scaling appropriately the stimuli beyond the range learned.

### Perceptual rules involved in size-concept learning

The apparatus used in our experiments did not allow for a precise control of the distance at which a decision (a stimulus choice) was made by a flying bee. Contrarily to other set-ups which have been conceived to this end (e.g., the Y-maze; see Giurfa et al., 1996) the rotating screen allows for decisions to be made at variable distances. However, we noticed that in our experiments, bees overflew the four stimuli presented at a time successively and only when they were relatively close to a given stimulus (approx. 6 cm) did they decide to land or not on the stimulus. Their choice operated therefore on the basis of a successive rather than simultaneous comparison. The bees may have then succeeded in the task by comparing at any time in the same foraging bout, i.e., at any stimulus view, the previous size they had acquired with the actually perceived size and decide to land if this size is equal or larger (group trained to the concept “larger than”) or equal or smaller (group trained to the concept “smaller than”) than the stored previous stimulus size. This scenario is particularly interesting, not only because it mimics a natural foraging situation in which bees perform successive floral choices, but also because it suggests that the decision rule may be based on a working memory that is employed successively within a foraging bout and that can be readjusted from bout to bout. The criterion used by the bees to define the viewing distance remains to be determined but this may be linked to the amount of image expansion elicited by the whole setup (Baird et al., 2013). In addition with the size rule extraction, the bees had indeed to control for the stimuli viewing distance to allow relevant size comparison.

### The role of L-receptor contrast

The finding that achromatic L-photoreceptor contrast is necessary for extracting size relations (Figure 3) is consistent with previous works on bee spatial vision demonstrating the implication of this achromatic cue in visual stimulus detection and recognition (Giger and Srinivasan, 1996; Hempel de Ibarra and Giurfa, 2003; Stach et al., 2004). Vision mediated by the L-receptor channel is known to have a comparatively finer resolution (Giurfa and Vorobyev, 1998) due to the quantitative dominance of green receptors in the ommatidia and their higher processing speed (Giurfa et al., 1996; Wakakuwa et al., 2005; Skorupski and Chittka, 2010). The fact that L-receptor contrast is required for relative size extraction explains why changing the color of stimuli from the training to the test (second transfer test) did not disturb the choice of bees as long as this cue was available; it is probable that bees focused primarily on L-receptor contrast because it allows determining stimulus contour and thus provides an estimation of stimulus size (Hempel de Ibarra and Giurfa, 2003).

In other animals, including vertebrates from remarkably different environments, there is also evidence of different photoreceptor signal processing underlying specific visual functions (Livingstone and Hubel, 1988; Krauss and Neumeyer, 2003; Neumeyer, 2003; Jones and Osorio, 2004; Bhagavatula et al., 2009; Hunt et al., 2009) suggesting that specialization for processing specific wavelength receptors for certain visual tasks may be a wide spread solution for visual processing in the animal world.

### Evolutionary and ecological scenarios for conceptual learning

Our results confirm that directly linking cognitive abilities to brain size is not appropriate: whilst larger brain size certainly enables parallel and efficient information processing and increased memory capacities (Roth and Dicke, 2005), cognitive skills do not necessarily require large and complex brains (Chittka and Niven, 2009; Chittka and Skorupski, 2011; Avarguès-Weber et al., 2011a; Avarguès-Weber and Giurfa, 2013). Evolutionary constraints on brain size in some insects like honeybees may have favored computational efficiency (Chittka and Skorupski, 2011). So far, evidence of concept learning is mostly restricted to large brained animals such as primates and dolphins but this may simply reflect a bias to presuppose cognitive abilities to be limited to such animals (Chittka and Niven, 2009; Avarguès-Weber and Giurfa, 2013; Manger, 2013). Indeed, many species share with honeybees an ecological context in which concept manipulation would be beneficial, for instance in the scenario of a central-place navigator that has to return always to the same location in space and needs to extract relations between landmarks to set efficient navigation strategies (Avarguès-Weber and Giurfa, 2013).

One recent study compared relational learning in both honeybees and the stingless bee *Melipona rufiventris*, and showed interestingly that whilst honeybees can learn arbitrary relations using delayed matching to sample visual problems, the stingless bees could not learn to solve problems this way (Moreno et al., 2012), thus suggesting restrictive evolutionary conditions for relational learning to occur. This difference makes sense as rather than using spatial visual learning as honey bees, most stingless bees (Apidae: Meliponini) mark with scent the pathways to the foraging sources (see Nieh, 2004 for review).

The current findings are likely to be of high value for interpretations of information extracted by bees when interacting with different flowers in complex natural environments. The cues that have typically been associated with reliable flower recognition at a distance include color, shape, symmetry and olfaction, but previously it was not suspected that an insect with a small brain could learn relationship rules to use relative size as a reliable identifying feature of flowers. This faculty could allow bees to counter mimic or invasive flower species strategy that exploit natural bee’s attraction for larger flowers and therefore develop flowers that are usually larger than those of the original species (Martin, 2004; Naug and Arathi, 2007; Schaefer and Ruxton, 2009). We demonstrate indeed that bees are able to repress their tendency to land on larger flowers and reliably choose the rewarding smaller flowers. The relative size of stimuli could thus be used for decision making as information by itself or even in concordance with spatial information to build configurational representations of objects for either foraging or navigational purposes. Our study thus provides new insights into conceptual learning in honeybees and confirms the bee as a promising and accessible model to unravel neurobiological mechanisms and ecological conditions for conceptual abilities to evolve.

## Authors contributions

Aurore Avarguès-Weber, Daniele d’Amaro and Adrian G. Dyer designed the experiments. Daniele d’Amaro, Marita Metzler and Adrian G. Dyer performed the experiments. Aurore Avarguès-Weber, Daniele d’Amaro and Marita Metzler analyzed the data. Aurore Avarguès-Weber and Adrian G. Dyer wrote the manuscript.

## Conflict of interest statement

The authors declare that the research was conducted in the absence of any commercial or financial relationships that could be construed as a potential conflict of interest.
